# Extracellular vesicles containing GAS6 protect the liver from ischemia-reperfusion injury by enhancing macrophage efferocytosis via MerTK-ERK-COX2 signaling

**DOI:** 10.1038/s41420-024-02169-y

**Published:** 2024-09-10

**Authors:** Longyu Miao, Chaoqun Yu, Ge Guan, Xiaoyu Luan, Xiaoshuang Jin, Meiqi Pan, Yuzhen Yang, Jiaoyang Yan, Peng Chen, Guohu Di

**Affiliations:** 1 Department of Special Medicine, School of Basic Medicine, Qingdao University, Qingdao, Shandong China; 2 Organ Transplantation Center, The Affiliated Hospital of Qingdao University, Qingdao, Shandong China; 3 Department of Clinical Medicine, Qingdao Medical College, Qingdao University, Qingdao, Shandong China; 4 Department of Clinical Medicine, Xinjiang Medical University, Urumqi City, Xinjiang Uygur Autonomous Region China; 5 Institute of Stem Cell and Regenerative Medicine, School of Basic Medicine, Qingdao University, Qingdao, Shandong China; 6 Department of Thoracic Surgery, Qingdao Eighth People’s Hospital, Qingdao, Shandong China

**Keywords:** Mesenchymal stem cells, Protein-protein interaction networks, Liver fibrosis

## Abstract

Hepatic ischemia-reperfusion injury (HIRI) is a significant issue during liver transplantation and surgery, contributing to the liver failure or even mortality. Although extracellular vesicles derived from mesenchymal stem cells (MSC-EVs) have shown substantial potentials in cell replacement therapy of various organ ischemia reperfusion injuries (IRIs), the precise mechanisms remain unclear. In this study, we demonstrate that systemic MSC-EVs administration is predominantly absorbed by macrophages, and verified that it could significantly reduce the liver injury and inflammatory response in mice suffering from HIRI. Furthermore, treatment with MSC-EVs induces macrophage polarization toward an anti-inflammatory phenotype. Mechanistically, proteomic profiling reveals an enrichment of growth arrest-specific 6 (GAS6) in MSC-EVs, significantly promoting the activation of myeloid-epithelial-reproductive tyrosine kinase/extracellular regulated protein kinases/cyclooxygenase 2 (MerTK/ERK/COX2) signaling pathway in macrophages and further enhancing their efferocytosis efficiency. Knockdown of GAS6 via lentiviral transfection or inhibition of MerTK using UNC2025 (a MerTK small molecule inhibitor) partially eliminates the protective effects of MSC-EVs on macrophage efferocytosis and liver injury. Overall, our findings support that MSC-EVs enriched GAS6 execute an anti-inflammation effect, highlighting that treatment based on the modulation of macrophage function by MSC-EVs as a promising approach in IRI.

**Figure:**
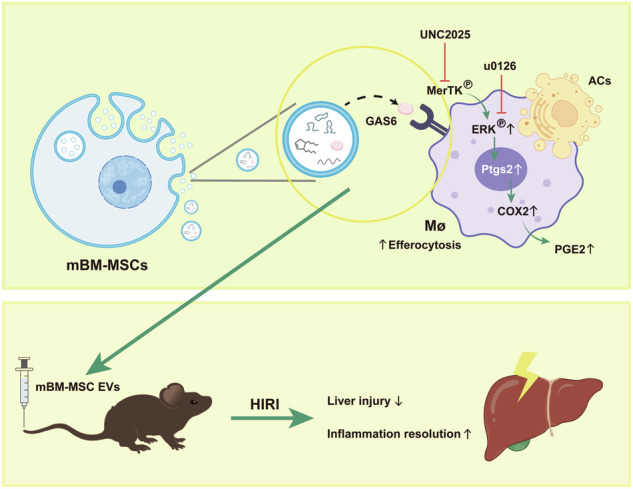
HIRI is a thorny problem after liver surgery such as liver transplantation. In a murine model of HIRI, MSC-EVs enriched GAS6 effectively enhance macrophage efferocytosis both in vivo and in vitro through the GAS6/MerTK/ERK/COX2 signaling pathway and significantly mitigate liver injury. This image was drawn by the authors.

HIRI is a thorny problem after liver surgery such as liver transplantation. In a murine model of HIRI, MSC-EVs enriched GAS6 effectively enhance macrophage efferocytosis both in vivo and in vitro through the GAS6/MerTK/ERK/COX2 signaling pathway and significantly mitigate liver injury. This image was drawn by the authors.

## Introduction

Although significant progress has been made in the study of hepatic ischemia/reperfusion injury (HIRI), it remains an inevitable pathophysiological process and a major clinical challenge during liver transplantation and other liver surgeries [1–3]. HIRI encompasses two interconnected phases: local ischemic insult and oxygen-free radical-induced inflammatory reperfusion injury. During the initial phase, glycogen consumption, oxygen deprivation, and adenosine triphosphate depletion lead to hepatocyte death from ischemia injury [4], while the subsequent reperfusion phase is characterized by intensified innate immunity and sterile inflammatory response [5]. Macrophages and neutrophils mediated sterile inflammatory response serves as the primary cause of organ damage [6]. Therefore, discovering more effective approaches to mitigate HIRI hold immense clinical significance.

Efferocytosis is defined as the process by which phagocytes clear dead cells [7]. Among them, macrophages, as the primary category of phagocytes, play a crucial role in the pathogenesis of HIRI [8]. Apart from recruiting host immune cells, they actively promoted inflammation resolution and tissue repair through efferocytosis. This function relies on the recognition of eat-me signals on apoptotic cell surfaces by macrophage receptors such as myeloid-epithelial-reproductive tyrosine kinase (MerTK). Additionally, bridging molecules like growth arrest-specific 6 (GAS6) or milk fat globule epidermal growth factor-factor VIII facilitate efficient removal of dying cells [9–11]. Recent research has highlighted how GAS6-Mer interaction can offer both anti-inflammatory and pro-survival effects against HIRI [12–14], underscoring the significance of targeting phagocytic signaling for mitigating liver injury.

Mesenchymal stem cells (MSCs) are a group of adult stem cells that possess regenerative potential, multipotent capacity, and immunoregulatory effects, making them highly attractive of tissue repair [15, 16]. Accumulating studies have demonstrated that MSCs exert their immunomodulatory functions through extracellular vesicles (EVs), which are submicron structures secreted by cells containing proteins, nucleic acids, and lipids [17–19]. Moreover, EVs derived from bone marrow MSCs (MSC-EVs) have the capability to mediate and regulate various cellular processes in target cells by transmitting their cargo via receptor-ligand interactions, endocytosis or direct fusion with the cell membrane [20, 21]. Although the protective effect of MSC-EVs against HIRI has been confirmed [22–24], the precise regulatory mechanism remains unknown.

In the present study, we demonstrated that MSC-EVs were enriched in GAS6 and elucidated their role in promoting M2 macrophage polarization via enhancing macrophage efferocytosis in the liver. This process triggered rapid clearance of apoptotic cells and regulated the restoration of liver homeostasis after HIRI.

## Results

### Isolation, characterization, and in vivo distribution of EVs derived from mouse bone marrow MSCs (mBM-MSC-EVs)

**Fig. 1:**
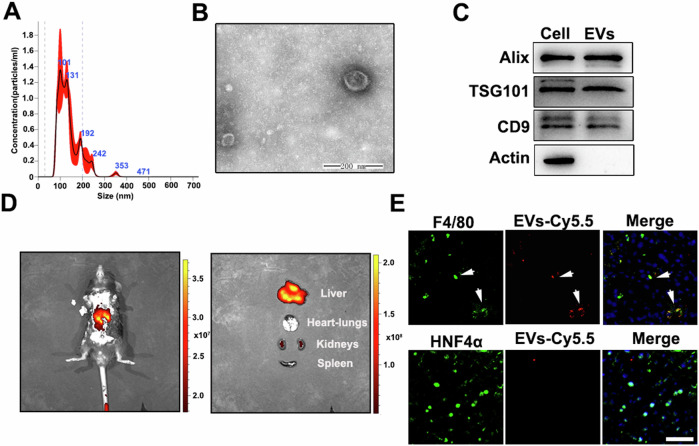
Characterization and in vivo distribution of MSC-EVs. The characterization of MSC-EVs by (**A**) particle size (**B**), TEM, and (**C**) Western blotting analysis. **D** The biodistribution of Cy5.5-labeled MSC-EVs was assessed following intravenous administration after ischemia surgery. Representative IVIS images of mice injected with MSC-EVs were obtained. **E** Fluorescence staining of EVs-Cy5.5, F4/80 or HNF4α in the murine liver following HIRI, Scale bars = 50 µm.

MSCs were isolated and characterized by cell surface marker detection, and multilineage differentiation potential tests. MSC-EVs were isolated as our previous description and detected using a particle analyzer. The nanoparticle tracking analysis (NTA) results showed that the size was concentrated in the range of 100–200 nm (Fig. 1A). Moreover, transmission electron microscope (TEM) image displayed MSC-EVs with a typical cup-shaped morphology (Fig. 1B). Additionally, the surface markers of Alix, TSG101, CD9 were identified by immunoblot analysis which met the identification criteria of EVs (Fig. 1C).

To further investigate the distribution of MSC-EVs in mice with HIRI, we constructed the 70% HIRI mice models, followed by administering Cy5.5-stained MSC-EVs via tail vein after the restoration of blood supply. At 6 h post-surgery, mice were subjected to observation using bioluminescent in vivo imaging system (IVIS). The results demonstrated that Cy5.5 fluorescence predominantly accumulated in the injured liver of mice (Fig. 1D). Following this, euthanasia was performed and liver tissue was collected for subsequent experiments. Macrophages and hepatocytes were specifically labeled with F4/80 and Hepatocyte Nuclear Factor 4 Alpha (HNF4α) markers, respectively. Co-localization between EVs-Cy5.5 and F4/80 was observed (Fig. 1E), suggesting preferential uptake of MSC-EVs by hepatic macrophages rather than hepatocytes. Also, flow cytometry analysis showed that 22.7 ± 1.4% F4/80 positive macrophages (F4/80^+^ cells) captured EVs-Cy5.5, while in F4/80 negative cells (F4/80^-^ cells), only 0.44 ± 0.05% cells uptaking EVs-Cy5.5 (Fig. S1A, B).

### MSC-EVs treatment has no long-term effects on liver fibrosis

In order to investigate the impact of MSC-EVs on hepatic fibrosis, HIRI mice were intravenously injected with MSC-EVs or PBS and sacrificed 7 days after surgery. Histological analyses including H&E staining, sirius red staining, and Alpha-smooth muscle actin (α-SMA) immunohistochemistry revealed no significant differences between the groups treated with MSC-EVs or PBS (Fig. S2A–C). Additionally, examination of fibrogenic gene expression such as transforming growth factor-β (TGF-β), collagen type I alpha 1 chain (COL1A1), α-SMA, showed no significant differences between the two groups (Fig. S2D), which was further confirmed by α-SMA immunoblot analysis (Fig. S2E).

### MSC-EVs treatment alleviated liver injury after HIRI

**Fig. 2:**
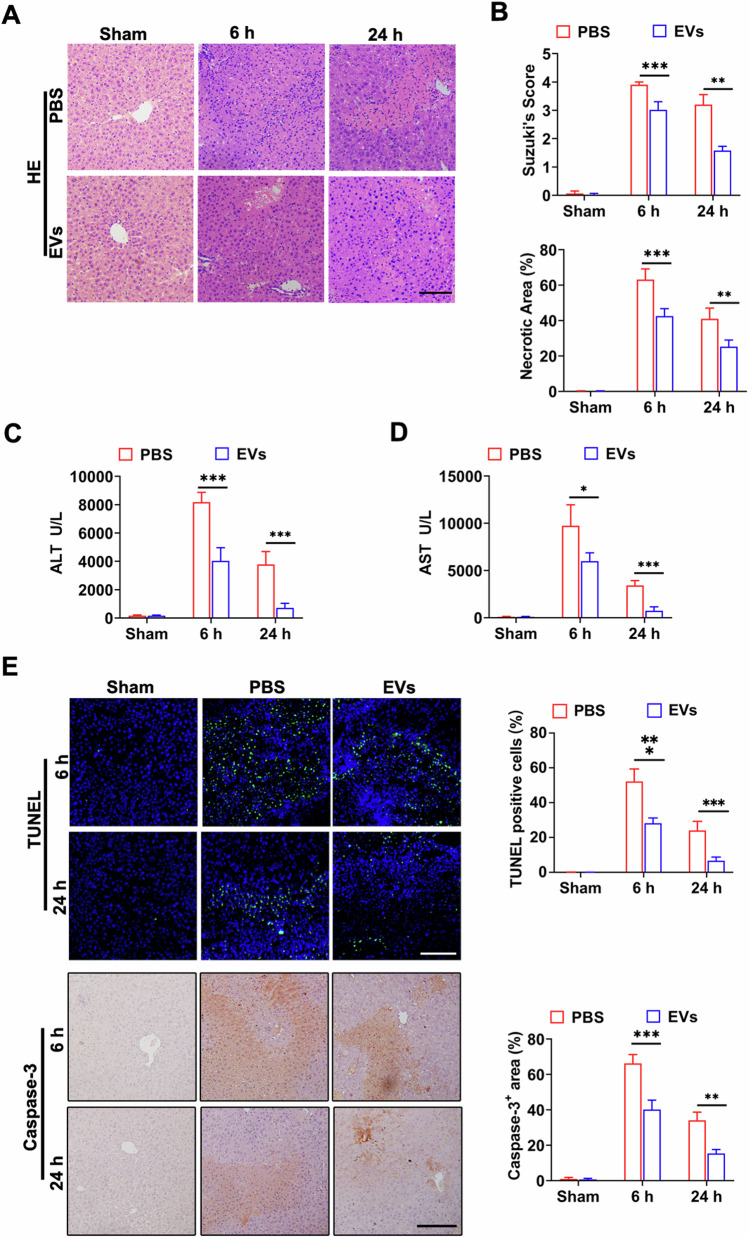
The systematic administration of MSC-EVs mitigates HIRI. MSC-EVs or PBS were administered intravenously immediately after hepatic ischemia for 60 min, followed by reperfusion for up to 6 h or 24 h. Representative images of liver tissue stained with H&E (**A**), Suzuki’s score and hepatocyte necrosis (**B**) in each group were obtained (*n* = 5 per group), Scale bars = 100 µm. Serum ALT (**C**) and AST (**D**) levels were measured (*n* = 5 per group). **E** Cell apoptosis was assessed using TUNEL assays (upper) and immunostaining of Caspase-3 (lower). The number of TUNEL-positive cells and Caspase-3-positive cells was quantified (*n* = 5 per group), Scale bars = 100 µm. Data were presented as the mean ± SD. *P < 0.05, **P < 0.01, ***P < 0.001.

To evaluate the therapeutic effects of MSC-EVs on HIRI, we initially established a mouse model with 60 min of warm ischemia followed by either 6 or 24 h of reperfusion, as our previous description [25]. Histological analysis using hematoxylin-eosin staining (H&E) staining demonstrated significant loss of hepatocyte integrity, extensive hepatocyte necrosis, and inflammatory response in the phosphate buffer saline (PBS) group. In contrast, mice pre-treated with MSC-EVs exhibited reduced hepatocyte damage (Fig. 2A). Furthermore, levels of Suzuki scores, hepatocyte necrosis, and ALT/AST were significantly decreased in the MSC-EVs treated group compared to the vehicle control (Fig. 2B–D), indicating that MSC-EVs effectively mitigated liver injuries. To further investigate the mitigation of hepatocyte death by MSC-EVs, we evaluated hepatic cell apoptosis through TUNEL staining and—analysis. HIRI induced hepatocyte apoptosis and upregulated Caspase-3 expression, while administration of MSC-EVs significantly attenuated hepatic apoptosis compared to the mice in the PBS group (Fig. 2E).

### MSC-EVs alleviated inflammatory response promoted reparative macrophage phenotype after HIRI

**Fig. 3:**
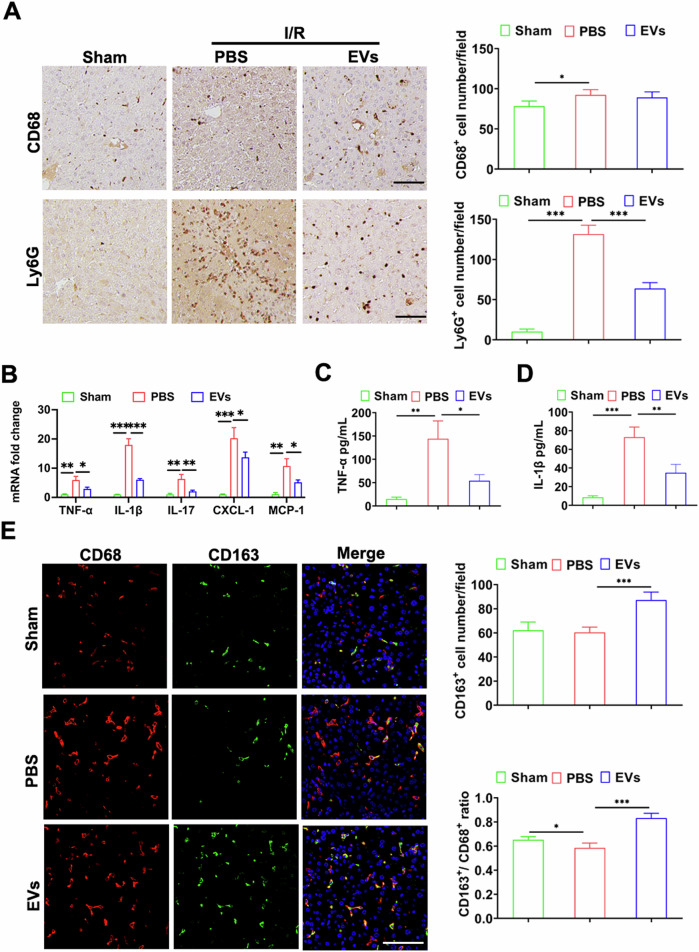
MSC-EVs alleviated inflammatory response promoted reparative macrophage phenotype after HIRI. **A** Representative immunohistochemistry images of CD68 and Ly6G in each group after 6 h of reperfusion. The quantitative analysis was performed to determine the number of CD68+ cells and Ly6G+ cells in the immunohistochemistry samples (*n* = 5 per group), Scale bar = 100 µm. **B** The mRNA expression levels of TNF-α, IL-1β, IL-17, CXCL-1, and MCP-1 were measured in liver tissue 6 h post-reperfusion (*n* = 5 per group). **C**, **D** Serum levels of TNF-α and IL-1β were quantified using ELISA (*n* = 5 per group). **E** Representative fluorescence staining images showing the presence of CD68 and CD163 in sham, PBS, MSC-EVs groups 6 h after reperfusion. The quantitative analysis was conducted to determine the number of CD68^+^ cells and CD163^+^ cells in the fluorescence staining samples, Scale bar = 50 µm. Data were presented as the mean ± SD. *P < 0.05, **P < 0.01, ***P < 0.001.

HIRI is characterized by immune cell infiltration and a cascade of inflammatory reactions. Immunohistochemistry staining results revealed significantly enhanced infiltration of Ly6G^+^ neutrophils at 6 h after reperfusion. Mice injected with MSC-EVs exhibited considerably less infiltration compared to mice injected with PBS. While there was no difference in the number of CD68^+^ macrophages between the two groups (Fig. 3A). Furthermore, livers treated with MSC-EVs showed significantly lower expression levels of proinflammatory genes tumor necrosis factor alpha (TNF-α), interleukin-1β (IL-1β), interleukin-17 (IL-17), and chemokines C-X-C motif chemokine ligand (CXCL1) and monocyte chemoattractant protein-1 (MCP-1) compared to those in the PBS group (Fig. 3B). Moreover, enzyme-linked immunosorbent assay (ELISA) results revealed a significant reduction in serum TNF-α and IL-1β levels in the MSC-EVs treated group (Fig. 3C, D). In summary, these findings suggest that treatment with MSC-EVs effectively mitigated hepatic inflammatory response.

To further elucidate the mechanism underlying liver injury repaired by MSC-EVs, we performed immunofluorescence co-staining of CD68 and CD163 on liver tissue to identify double-positive M2 hepatic macrophages. In comparison to the PBS group, there was a significant increase in the number of CD163^+^ cells and an elevated ratio of CD163^+^/CD68^+^ cells in the MSC-EVs group. These findings suggested that MSC-EV administration induces polarization of macrophages towards an inflammation-suppressive M2 phenotype (Fig. 3E).

### Proteomic profiling identified GAS6 enrichment in extracellular vesicles

**Fig. 4:**
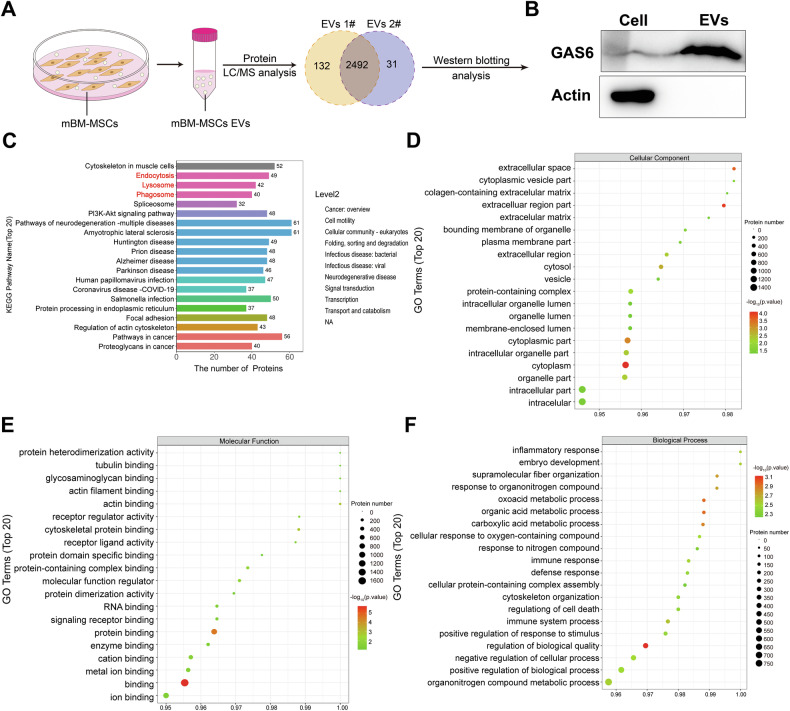
Identification of MSC-EVs protein cargo that influences the phagocytosis of cells. **A** LC-MS/MS (nanoLC-QE) proteomic analysis for MSC-EVs. **B** Western blot analysis of GAS6 in cells and EVs. **C** KEGG pathway analysis of the MSC-EVs. **D**–**F** Gene ontology (GO) enrichment analysis of the MSC-EVs.

The protein components of MSC-EVs were identified through Liquid Chromatograph Mass Spectrometer (LC-MS/MS, nanoLC-QE) analysis. Two independent preparations of MSC-EVs revealed a total of 2492 common proteins, indicating the homogeneity of our extracted samples (Fig. 4A). Importantly, GAS6 was found to be enriched in MSC-EVs compared to cell (Fig. 4B), which showed strong association with efferocytosis, and modulated the phenotype of hepatic macrophages. Furthermore, kyoto encyclopedia of genes and genomes (KEGG) pathway analysis further demonstrated a large number of proteins associated with endocytosis, lysosome, and phagosome within MSC-EVs (Fig. 4C). Gene ontology (GO) enrichment analysis revealed the presence of proteins associated with various biological processes (BP), cellular compartments (CC), and molecular functions (MF) within MSC-EVs as depicted in Fig. 4D-F.

### MSC-EVs promoted macrophage efferocytosis through activation of the MerTK/ERK/COX2 signaling pathway

**Fig. 5:**
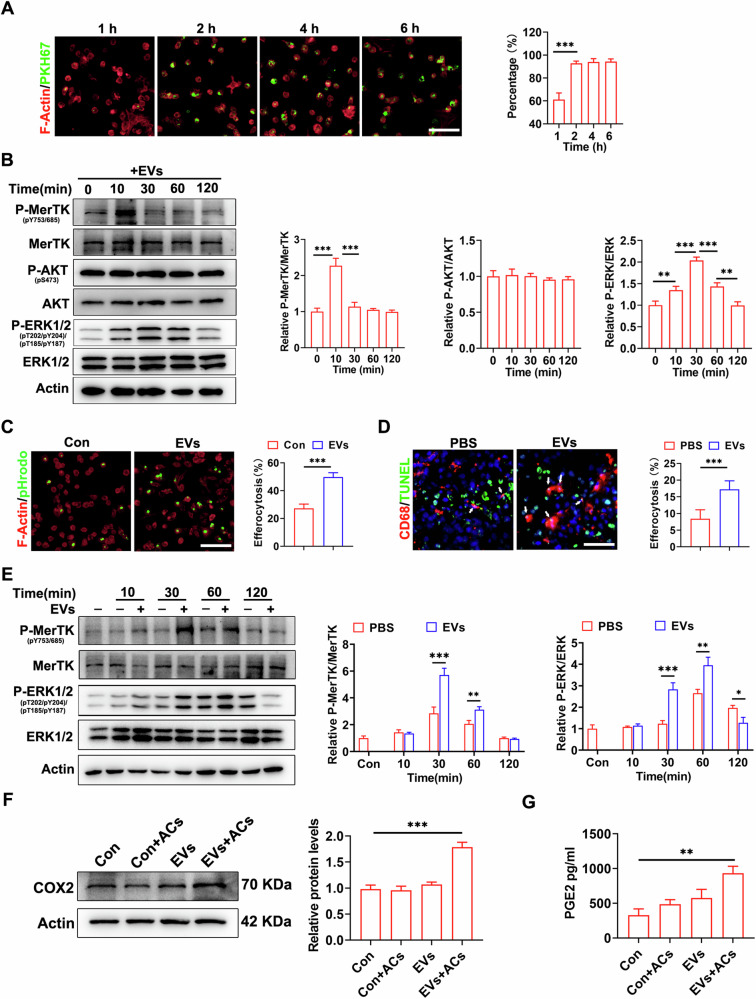
MSC-EVs promotes macrophage efferocytosis through activation of the MerTK/ERK/COX2 signaling pathway. **A** BMDMs labeled with F-Actin (red) were incubated with apoptotic neutrophils labeled with PKH67 (green), and the uptake efficiency was assessed, 200–300 cells in each field were quantified, Scale bar = 50 µm. **B** BMDMs were incubated with MSC-EVs for indicated times, and the expression of p-MerTK, MerTK, p-AKT, AKT, p-ERK, ERK in BMDMs was detected by western blot. **C** BMDMs were pretreated with MSC-EVs or PBS control before efferocytosis. Then, BMDMs labeled with F-Actin (red) were incubated with apoptotic cells labeled with pHrodo (green) to assess efferocytosis, 200–300 cells in each field were quantified, Scale bar = 50 µm. **D** Mice were subjected to HIRI after being pretreated with MSC-EVs or PBS, representative CD68 and TUNEL staining of liver sections were performed (*n* = 5 per group), in situ efferocytosis was marked by white arrows, 300–500 cells in each field were quantified, Scale bar = 50 µm. **E** BMDMs pretreated with MSC-EVs showed altered expression levels of p-MerTK, MerTK, p-ERK,and ERK when co-incubated with apoptotic neutrophils as detected by western blot analysis. **F** BMDMs were pre-treated with MSC-EVs for 2 h, followed by incubation with (+) or without (-) ACs for 45 min. After an additional 6 h of incubation, COX2 protein expression was assessed in the cells using western blot analysis. (**G**) PGE2 production in cultural supernatant were assessed by ELISA. Data were presented as the mean ± SD. *P < 0.05, **P < 0.01, ***P < 0.001.

To assess the absorption efficiency of macrophages, we labeled MSC-EVs with green fluorescent PKH67 and subsequently incubated them with bone marrow-derived macrophages (BMDMs) marked with F-Actin for varying duration (1 h, 2 h, 4 h, 6 h). After a 2-hour incubation, it was observed that macrophages phagocytosed about 90% of the EVs, indicating rapid engulfment by MSC-EVs (Fig. 5A). Furthermore, to validate the downstream signaling pathway of GAS6, MerTK/ERK signaling pathway was found to be activated in the group pretreated with MSC-EVs; however, no significant impact on the serine/threonine-specific protein kinase (AKT) signaling pathway was observed (Fig. 5B), macrophage efferocytosis was assessed by co-incubating BMDMs, with apoptotic cells (ACs) stained with pHrodo dye. As expected, pretreatment of MSC-EVs enhanced efferocytosis of ACs by macrophages (Fig. 5C). Moreover, in situ efferocytosis was achieved by quantifying the co-localization of TUNEL-positive ACs and CD68 positive macrophages after HIRI. Consistent with our findings, administration of MSC-EVs resulted in a significant enhancement of efferocytosis efficiency by macrophages in situ (Fig. 5D). Furthermore, MSC-EVs induced phosphorylation of MerTK and ERK in BMDMs in the presence of ACs (Fig. 5E), as well as cyclooxygenase 2 (COX2) expression and prostaglandin e2 (PGE2) production (Fig. 5F, G).

### GAS6 silence reversed the efficacy of MSC-EVs on macrophage efferocytosis and HIRI

**Fig. 6:**
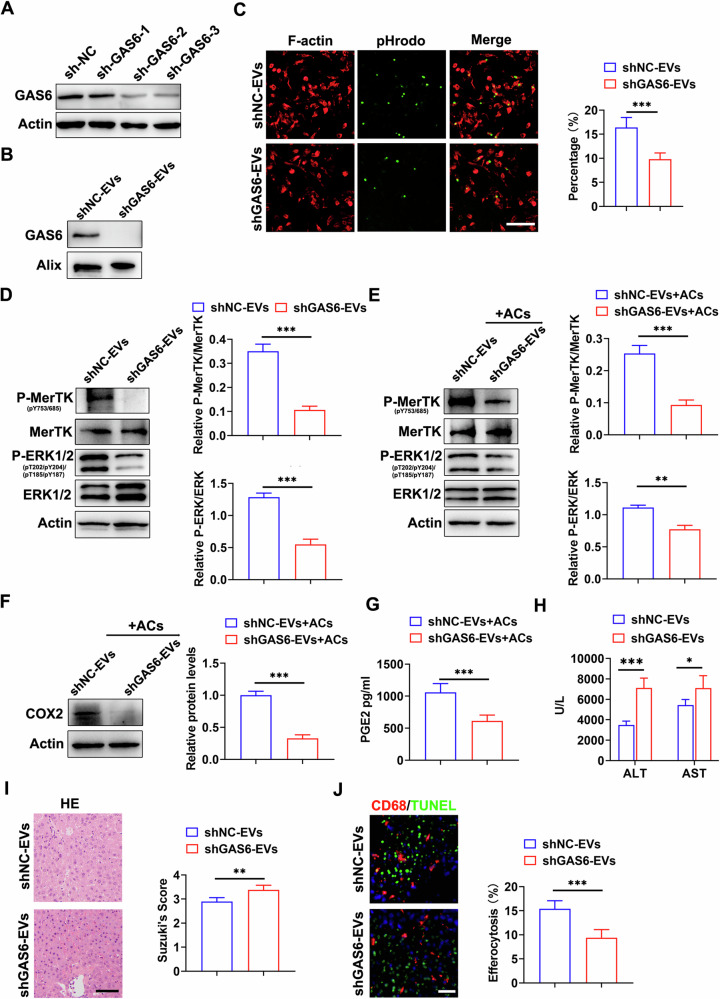
GAS6 silence reversed the efficacy of MSC-EVs on macrophage efferocytosis and HIRI. BM-MSCs were transfected with GAS6 lentivirus or Negative Control lentivirus. **A** Representative blots of MSCs after transfection with different sets of GAS6-specific Lentivirus. **B** Western blot showing efficient knockdown of GAS6 in MSC-EVs after 48 h transfection with GAS6-specific Lentivirus. **C** BMDMs pretreated with shNC-EVs or shGAS6-EVs were labeled with F-Actin (red) and incubated with apoptotic cells labeled with pHrodo (green), efferocytosis was assessed, 200–300 cells in each field were quantified, Scale bar = 50 µm. **D** The levels of p-MerTK and p-ERK1/2 in BMDMs were determined by Western blotting after treatment with shNC-EVs or shGAS6-EVs. **E** The expression of p-MerTK and p-ERK1/2 in BMDMs pretreated with shNC-EVs or shGAS6-EVs and incubated with apoptotic cells was detected by Western blot analysis. **F** Western blot results demonstrated the expression of COX2. **G** PGE2 levels in cell culture supernatant were measured using ELISA. **H** Assessment of serum ALT and AST levels. **I** Representative liver histology image and Suzuki’s score quantification, Scale bar = 100 µm. **J** In situ assessment and quantification of efferocytosis, 300–500 cells in each field were quantified, Scale bar = 50 µm. Data were presented as the mean ± SD. *P < 0.05, **P < 0.01, ***P < 0.001.

To confirm that the promotion of macrophage efferocytosis by MSC-EVs was mediated by GAS6, we performed GAS6 knockdown through transfection with GAS6-specific lentivirus. The knockdown efficiency of sh-GAS6 was verified via western blotting (Fig. 6A, B). Compared to shNC-EVs, shGAS6-EVs exhibited a reduced ability to promote efferocytosis of ACs (Fig. 6C). Furthermore, the activation of the MerTK/ERK/COX2 pathway was significantly diminished in BMDMs treated with shGAS6-EVs, both with or without coculture with ACs (Fig. 6D, F). Additionally, pretreatment with shGAS6-EVs led to a decrease in PGE2 production by BMDMs compared to shNC-EVs (Fig. 6G). In vivo experiments demonstrated that the protective effect of MSC-EVs on liver injury was largely abolished upon knockdown of GAS6 (Fig. 6H, I), as well as macrophage efferocytosis in situ (Fig. 6J). Collectively, these findings suggest that GAS6 played a crucial role in mediating MSC-EVs-induced promotion of BMDM efferocytosis and liver injury following ischemia-reperfusion injury.

### MerTK and ERK inhibition reversed the efficacy of MSC-EVs on macrophage efferocytosis and HIRI

**Fig. 7:**
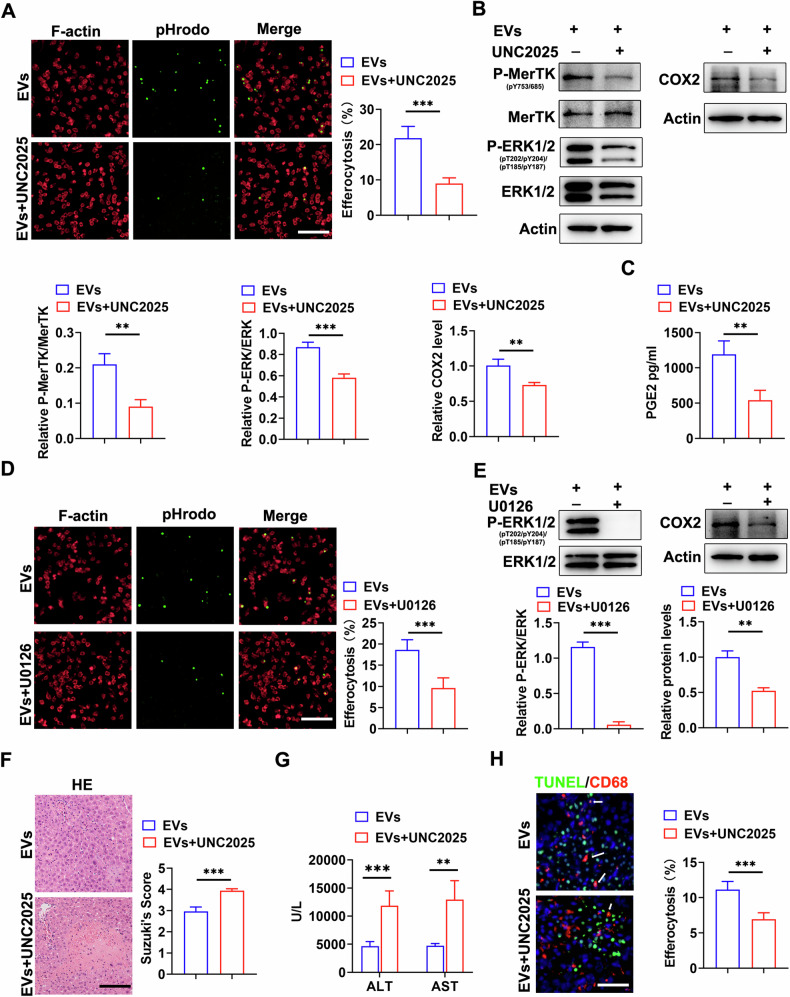
MerTK and ERK inhibition reversed the efficacy of MSC-EVs on macrophage efferocytosis and HIRI. **A** Representative immunofluorescence images depicting efferocytosis of ACs (green) by BMDMs (red), pretreated with or without UNC2025, in the presence of MSC-EVs, and quantified, Scale bar = 50 µm. **B** Quantification of p-MerTK, p-ERK1/2, and COX2 levels in BMDMs pretreated with or without UNC2025 in the presence of MSC-EVs. **C** Measurement of PGE2 levels in the cell culture supernatant using ELISA. **D** Quantitative analysis of efferocytosis performed, Scale bar = 50 µm. **E** Evaluation of p-ERK, ERK and COX2 expression levels in BMDMs treated with or without the ERK inhibitor U0126 in the presence of MSC-EVs. **F** Assessment of serum ALT and AST levels. **G** Representative liver histology image and Suzuki’s score quantification, Scale bar = 100 µm. **H** In situ assessment and quantification of efferocytosis, Scale bar = 50 µm. Data were presented as the mean ± SD. *P < 0.05, **P < 0.01, ***P < 0.001.

The MerTK-specific inhibitor UNC2025 was employed to investigate the dependence of MSC-EVs therapeutic effects on the MerTK/ERK pathway. Remarkably, UNC2025 treatment significantly impeded MSC-EV-induced phagocytosis of ACs by BMDMs (Fig. 7A). Furthermore, UNC2025 treatment effectively suppressed MSC-EVs-induced phosphorylation of MerTK and ERK, as well as COX2 expression in BMDMs (Fig. 7B). UNC2025 treatment also led to a notable reduction in PGE2 production (Fig. 7C). Similarly, U0126 treatment exhibited significant inhibition of MSC-EV-induced phagocytosis of ACs by BMDMs (Fig. 7D), along with decreased COX2 expression levels (Fig. 7E). Moreover, in vivo results demonstrated that administration of UNC2025 resulted in increased liver damage compared to the MSC-EVs group. This was evidenced by an augmented area of parenchymal necrosis and elevated serum alanine transaminase (ALT) and aspartate transaminase (AST) levels (Fig. 6F, G). TUNEL/CD68 immunofluorescence co-staining revealed that inhibition of MerTK activation markedly reduced macrophage efferocytosis in situ (Fig. 7H). Collectively, these findings strongly suggested that the efferocytic effects induced by MSC-EVs were reliant on activation of the MerTK/ERK pathway.

## Discussion

In this study, we provided novel and significant insights into the application of MSC-EVs. Our findings demonstrated that MSC-EVs can effectively protect against the HIRI in a murine model. Furthermore, systemic administration of MSC-EVs specifically targeted hepatic macrophages rather than hepatocytes, leading to not only the activation of M2 phenotype in macrophages but also an enhancement in their efferocytosis activity. Mechanistically, our protein array analysis revealed an enrichment of GAS6 in MSC-EVs and identified MerTK as its corresponding receptor. Finally, we elucidated that the activation of MerTK/ERK/COX2 signaling pathway through direct ligand-receptor binding may be involved in modulating macrophage efferocytosis and consequently mitigating HIRI.

As the predominant innate immune cell in the liver, macrophages play a pivotal role in maintaining inflammatory responses and tissue homeostasis. Macrophage infiltration and activation are both causative factors and characteristic events of HIRI [26–28]. The phenotype of macrophages can be influenced by the local microenvironment during disease progression, leading to their polarization into M1 or M2 macrophages. Studies have shown that diabetes/hyperglycemia specifically triggered S1P/S1PR3 signaling and exacerbated HIRI by facilitating M1 polarization and inhibiting M2 polarization [29]. Therefore, controlling the pro-inflammatory effects of macrophages is essential to prevent exacerbation of HIRI.

Previous research has demonstrated that MSCs effectively alleviated both functional impairment and pathological damage associated with IRI through the release of beneficial paracrine factors [13]. In this study, following treatment with MSC-EVs, there was no significant change in the number of infiltrated CD68^+^ macrophages compared to vehicle control groups at 6 h post-reperfusion. Interestingly, consistent with our group’s previous findings, MSC-EVs possessed unique immunomodulatory functions by promoting the polarization of macrophages toward an M2 phenotype (CD68^+^CD163^+^) [30]. Moreover, our study demonstrated that the transcription and protein expression of cytokines (TNF-α and IL-1β) and chemokines (CXCL-1 and MCP-1) in mice suffered HIRI were reduced after administration of MSC-EVs, indicating that MSC-EVs facilitated a switch in macrophage phenotype towards a reparative state under inflammatory conditions.

GAS6 is a vitamin K-dependent protein that participates in immune regulation, inflammation, and apoptosis by binding to the TAM receptor family (Tyro3, AXL, and Mer) [31]. While much of the literature suggested that MSC-EVs influenced disease outcomes through the transfer of microRNA, an earlier study by Toh et al. indicated that MSC exosome-derived proteins have the potential to modulate numerous biological processes associated with disease pathogenesis, tissue repair, and regeneration [32]. Importantly, perivascular cell-derived EVs contain GAS6, which activates the GAS6/Axl pathway in endothelial cells to promote tumor angiogenesis [33]. Indeed, pericytes have been identified as a source of MSCs precursors in vivo in multiple organs [34]. Consistent with previous report, our study clarified that GAS6 was enriched in MSC-EVs by proteomic profiling. Nevertheless, there is still a lack of understanding regarding the mechanisms involved in both EVs-GAS6 interaction with the MerTK receptor and EVs-GAS6-induced activation of downstream signaling pathway in BMDMs. Although the role of TAM receptors in phagocytosis and bacterial killing has been a subject of controversy [35], they are generally recognized to be involved in maintaining homeostasis and promoting the efferocytosis during the resolution phase of inflammation [36, 37]. Recent studies have shown that deletion of MerTK nearly abolished phagocytosis of ACs by macrophages, whereas deletion of Axl, Tyro3, or both reduces macrophage phagocytosis by approximately 50% [38]. Moreover, MerTK-expressing monocytes and macrophages represent an immune-modulatory pro-restorative cell population [39, 40].

In models of acute liver injury, Axl^-/-^ and MerTK^-/-^ mice demonstrated increased accumulation of ACs and more severe liver injury compared to their wild-type counterparts. This observation suggested that the process of efferocytosis, which involved the clearance of ACs, required MerTK [41]. In this study, EVs-GAS6 might be delivered to BMDMs through receptor-mediated endocytosis and subsequently recirculated to activate the MerTK pathway. To confirm that, the beneficial effects of MSC-EVs on macrophage efferocytosis were partially inhibited both in vivo and in vitro using a MerTK/ERK inhibitor. Additionally, knockdown of GAS6 using shRNA significantly diminished the effects of MSCs on macrophages. Collectively, our results indicate that GAS6 plays a role in enhancing phagocytic capacity of macrophages mediated by MSC-EVs through targeting the MerTK/ERK signaling pathway rather than AKT signaling pathway.

Fibrosis is the consequence of an excessive and abundant deposition of extracellular matrix resulting from recurrent tissue damage or dysregulation of the repair process, in which macrophages play a dual regulatory role [42]. In fact, reparative mechanisms such as efferocytosis are necessary for responding to damage, but they can be beneficial only in transient situations and harmful when excessive [43]. On one hand, efferocytosis inhibits inflammation and facilitates tissue repair during acute liver injury; on the other hand, continuous activation of this signaling pathway contributes to chronic inflammation leading to fibrosis and tumorigenesis by evoking hepatic stellate cells while inhibiting anti-tumor immunity. In our study, our findings reveal that MSC-EVs can enhance macrophage phagocytosis of ACs through temporary and effective activation of the MerTK/ERK pathway but have no effect on long-term development of liver fibrosis. Thus, as a highly promising, safe, and specific endogenous nanomedicine carrier, MSC-EVs hold broad application prospects in HIRI. Taken together, our data demonstrated the enrichment of GAS6 in MSC-EVs and its ability to activate the MerTK/ERK pathway in macrophages, leading to their polarization towards a pro-repair phenotype and enhanced efferocytosis. Consequently, this mechanism mitigated HIRI, highlighting the crucial role of GAS6 in IRI pathogenesis and providing valuable insights for clinical interventions targeting HIRI.

## Materials

### Mouse model of HIRI and treatment

Male C57BL/6 mice, 8–10 weeks old, were purchased from Beijing Hua Fukang Biotechnology Co.,Ltd. (Beijing, China). All animal experimental conform to the protocols approved by the Experimental Animal Ethics Committee of College of Medicine, Qingdao University (QDU-AEC-2023360). Briefly, the mice were fasted for 12 h before surgery and anesthetized with 2% isoflurane. A midline laparotomy was performed, the blood supply of portal vein and hepatic artery in mouse liver were blocked by non-invasive vascular clip. This results in a reduction in hepatic blood flow of about 70%. Immediately after blocking 70% of the hepatic blood flow, mice (*n* = 5 per group) were separated randomly into five groups: sham, I/R + Vehicle (PBS,200ul), I/R + MSC-EVs (2 × 10^10^ particles/body), I/R + UNC2025 (50 mg/kg) + MSC-EVs (2 × 10^10^ particles/body), sh-NC-MSC-EVs or sh-GAS6-MSC-EVs (2 × 10^10^ particles/body) [22]. Then the clip was released after approximately 60 min followed by 6, 24 h reperfusion. Liver tissue and blood specimens were collected following the reperfusion phase.

### Primary mouse BM-MSCs and BMDMs isolation and culture

Isolation and culture of mouse BM-derived MSCs were performed as our previous description [30]. Briefly, MSCs from femurs and tibias of 3–4 weeks old C57BL/6 mice were cultured in mouse bone marrow mesenchymal stem cell complete medium. Then, medium was changed every 2–3 days until the cells reached 80% confluence and then passaged. MSCs at the third and fourth passages were used for subsequent experiments.

For the culture of BMDMs, the femur and tibia bones marrow cavities of C57BL/6J mice (6–8 weeks) were flushed with PBS, which was then passed through a cell strainer. Freshly harvested cells were cultured for 7 days in high-glucose DMEM containing 10% FBS and 1% penicillin streptomycin solution and 10 ng/ml recombinant mouse macrophage colony-stimulating factor (M-CSF). Fully mature BMDMs on day 7 were used for subsequent assays. Cells were cultured in 5% CO_2_ incubator at 37 °C.

### Extracellular vesicles isolation and characterization

The characterization and quantification of MSC-EVs were determined by TEM analysis, Western blotting analysis, and NTA as previous description [44, 45]. To separate EVs, the third or fourth generation of cells were grown to confluence, washed 3 times with PBS and then incubated in serum-free media at 37 °C. After 24 h, We collected the supernatant of cell culture and the dead cells and debris in the medium were removed via centrifugation at 2000*g* for 20 min. Then, the supernatant filtered with 0.22μm filters. An Amicon Ultra-15 Centrifugal Filter Unit (100 kDa; Millipore, Billerica, USA) was used to concentrate medium. Finally, MSC-EVs were isolated from the concentrated supernatants using Exoquick-TC™ (System Biosciences, CA, USA) according to the manufacturer’s instruction.

### Extracellular vesicles labeling and tracking

As previously mentioned, to observe the distribution of EVs in HIRI mice, MSC-EVs were labeled with Cyanine5.5 NHS (Cy5.5, 1:1000, Lumiprobe, Florida, USA) [30]. Stained EVs were injected into mice via tail vein immediately after the restoration of blood supply. After 6 h of reperfusion, bioluminescence pictures were captured and processed using an IVIS Lumina XRMS III in vivo imaging system (PerkinElmer, Waltham, MA, USA)., MSC-EVs were stained with PKH67, and then incubated with BMDMs for various time (1 h, 2 h, 4 h or 6 h) to observe the efficiency of BMDMs in phagocytosis of MSC-EVs. The uptake of MSC-EVs by BMDMs was photographed with an inverted fluorescence microscope.

### Induction of apoptosis and labeling of neutrophil

Total neutrophils from mouse bone marrow were obtained using the Mouse Neutrophil Enrichment Kit from Solarbio Science & Technology Co., Ltd. (Beijing, China) according to the manufacturer’s specifications. This process routinely yielded a neutrophil population of 90% purity as assessed by flow cytometry for CD11b, Ly6G and Gr-1 (data not shown). For neutrophil apoptosis, cells were collected into centrifuge tubes and placed in a 55 °C water bath for half an hour. The cells were then rinsed and resuspended in complete DMEM before adding them to macrophages at a 5:1 ACs: macrophage ratio. For some experiments, ACs were stained with pHrodo dye (Thermo Fisher Scientific) as described by the manufacturer’s protocol.

### Efferocytosis assay

For in situ efferocytosis, the terminal deoxynucleotidyl transferase dUTP-biotin nick end labeling (TUNEL) assay from a commercially available kit (Yeasen) was used to identify hepatocyte apoptosis according to the manufacturer’s instructions. After sealed with 5% BSA for 60 min, liver tissues were incubated with anti-CD68 (Servicebio,1:200) for a whole night at 4 °C, and counterstained with DAPI. For efferocytosis, BMDMs were plated in 48-well tissue culture plates and pretreated with MSC-EVs for 2 h. Apoptotic neutrophils were labeled with pHrodo green succinimidyl ester (Thermo Fisher Scientific) at 37 °C for 45 min and washed twice with PBS. Then the above two prepared cells were co-incubated at a ratio of 5:1 (ACs/Mϕ, where Mϕ denotes BMDMs) for 2 h. Then, Nonadherent cells were washed off with PBS. And BMDMs were further stained with Actin-Tracker Red-555 (Beyotime). Images were taken using the fluorescence microscope to identify the uptake of labeled ACs and five randomized fields were selected from each group and quantified using Image J software. Efferocytosis was evaluated as the percentage of pHrodo-green positive cells in all Actin-Tracker Red-555 positive cells.

### Transduction of recombinant lentivirus

To obtain MSC-EV-shNC and MSC-EV-shGAS6, MSCs at passage 2 were seeded in cell culture flasks to reach 30–50% confluency, followed by transfection with NC shRNA or GAS6 shRNA using a commercial kit (General Biol, Anhui, China). After transfection for 24–48 h, the culture medium was replaced with fresh medium. Then 2ug/ml puromycin was added to selectively enrich shRNA-positive cells. Knockdown of GAS6 were further detected by western blotting.

### Flow cytometry (FCM) analysis

The sample were obtained from primary cells in the HIRI mice treated with MSC-EVs which stained with Cy5.5. After reperfusion 6 h, primary hepatocytes, kupffer cells (KCs) and liver mononuclear cells were isolated from mice that underwent euthanasia using a two-step collagenase perfusion technique. Single-cell suspensions were incubated with anti-F4/80 (1:100, eBioscience, San Diego, CA, USA), anti-CD11b (1:100, eBioscience, San Diego, CA, USA), anti-CD45(1:100, Biolegend), and cell sorting was performed using a flow cytometer (BD, Franklin Lakes, NJ, USA). Then the quantification data were analyzed using Flowjo program (Tree Star, Ashland, OR, USA).

### Proteomic analysis of MSC-EVs

Proteome profiling of MSC-EVs was performed by Suzhou PANOMIX Biomedical Tech Co. Ltd. Briefly, MSC-EV samples (50 μl, obtained from 100 ml MSC culture medium) were quantified and samples with 30 μg proteins were performed with SDS-PAGE separation, followed by filter-aided sample preparation (FASP digestion).

LC-MS/MS analysis was performed on a timsTOF Pro mass spectrometry (Bruker) that was coupled to Nanoelute (Bruker). The mass spectrometer was operated in positive ion mode. The peptides were loaded onto a C18-reversed phase analytical column (Thermo Scientific Easy Column, 25 cm long, 75μm inner diameter, 1.9μm resin) in 95% buffer A (0.1% Formic acid in water) and separated with a linear gradient of buffer B (99.9% acetonitrile and 0.1% Formic acid) at a flow rate of 300 nl/min. The MS raw data for each sample were combined and searched using the MaxQuant 1.6.14 software for identification and quantitation analysis. The following options were used for protein identification: Enzyme=trypsin, Fixed modification=Carbamidomethyl (C), Dynamical modifications = Oxidation (M), Max Missed Cleavages = 2.

### Immunohistochemistry (IHC)

To monitor the pathological changes in liver tissues, all samples were fixed with 4% PFA, blocked, and then treated with Caspase-3 (1:200, CST, Cat#9662S), alpha-smooth muscle actin(1:200, Abcam, Cat# ab5694) antibody overnight at 4 °C before visualization by color development with 3,3-diaminobenzidine (DAB) (Servicebio).

### Western blot analysis

Western blot was performed as previously reported. Briefly, protein samples were separated by 7.5% or 10% SDS-PAGE and transferred to PVDF membrane. Thereafter, membranes were blocked with 5% nonfat dry milk solution for 1 h and were incubated overnight at 4 °C with anti-Alix (1:1000, HUABIO, ET1705-74), anti-TSG101 (1:1000, HUABIO, ET1701-59), anti-CD9 (1:1000, HUABIO, Cat# ET1601-9), anti-GAS6 (1:1000, Abclonal, Cat# A8545), anti-AKT1/2/3 (1:2000, HUABIO, Cat# ET1609-51), anti-Phosopho-AKT (pS473) (1:2000, HUABIO, Cat# ET1607-73), anti-ERK1/2 (1:2000, HUABIO, Cat# ET1601-29), anti-Phosopho-ERK1(pT202/pY204)+ERK2(pT185/pY187)(1:2000, HUABIO, Cat# ET1610-13), anti-MerTK (1:500, Affinity Bioscience, Cat# DF7344), anti-Phosopho-MerTK (pY753/685) (1:500, Affinity Bioscience, Cat# AF8443), anti-COX2 (1:1000, HUABIO, Cat# ET1610-23), anti-α-SMA (1:1000, Abcam, Cat# ab5694), anti-β-Actin (1:1000, Abclonal, Cat# AC026). Subsequently, the membranes were incubated with the secondary antibody for 1 h. Then, the specific bands were visualized by an enhanced chemiluminescence reagent kit (Meilunbio). Finally, and signals were detected with an automatic chemiluminescence image analysis system (Tanon, Shanghai, China), and the expression levels of all target proteins were normalized to β-actin with ImageJ software (U.S. National Institutes of Health, Bethesda, MD, USA).

### Real‑time quantitative PCR (RT‑qPCR)

Total RNA was extracted from cells using FastPure Cell/Tissue Total RNA Isolation Kit (Vazyme, Nanjing, China), and reverse transcribed to cDNA using Prime-Script First Strand cDNA synthesis kit (TaKaRa, Dalian, China) according to the manufacturer’s instructions. RT-PCR was performed according to the standard operating procedures. The relative mRNA expression levels were normalized to housekeeping gene GAPDH. The relative expression of each mRNA was calculated using the 2-ΔΔCT method. All sequences of primer are as follows:

**Table:** 

Gene	Accession number	Primer sequences
m-GAPDH	NM_001289726.1	Forward: GCCACCCAGAAGACTGTGGAT
		Reverse: GGAAGGCCATGCCAGTGA
m-TNF-α	NM_013693.3	Forward: ACAAGGCTGCCCCGACTAC
		Reverse: TGGGCTCATACCAGGGTTTG
m-IL-1β	NM_008361.4	Forward: CTTTCCCGTGGACCTTCCA
		Reverse: CTCGGAGCCTGTAGTGCAGTT
m-IL-17	NM_010552.3	Forward: GACTCTCCACCGCAATGAAGAC
		Reverse: CTCTTCAGGACCAGGATCTCTTG
m-CXCL1	NM_008176.3	Forward: CGCTTCTCTGTGCAGCGCTGCTGCT
		Reverse: AAGCCTCGCGACCATTCTTGAGTC
m-MCP-1	NM_011333.3	Forward: CAGCAAGATGATCCCAATGAGTAG
		Reverse: TTTTTAATGTATGTCTGGACCCATTC
m-TGF-β	NM_011577.2	Forward: CAACAATTCCTGGCGTTACCTT
		Reverse: CAAGAGCAGTGAGCGCTGAA
m-COL1A1	NM_007742.4	Forward: TGACTGGAAGAGCGGAGAGTACT
		Reverse: TTCGGGCTGATGTACCAGTTC
m-α-SMA	NM_007392.3	Forward: TGCCGAGCGTGAGATTGTC
		Reverse: CGTTCGTTTCCAATGGTGATC
m-Ptgs2	NM_011198.5	Forward: CAACTCCATCCTCCTGGAACA
		Reverse: TATTTCATCTCTCTGCTCTGGTCAA

### Statistical analysis

In this study, all data were obtained from at least three independent experiments. Statistical analysis were performed using Prism 8.0 software (GraphPad, San Diego, CA, USA). Unpaired Student’s *t* test was used to determine differences between the two groups. A nonparametric test was used when the data did not meet the normal distribution and homogeneity of variance assumptions. Results were presented as means ± SD and differences with *P* values smaller than 0.05 were considered statistically significant. *P < 0.05; **P < 0.01, ***P < 0.001.

## Supplementary information

Supplementary figures and figure legends

## Data Availability

The data that support the findings of this study are available from the corresponding author upon reasonable request.
