# Supplementary material for: Extracellular vesicles containing GAS6 protect the liver from ischemia-reperfusion injury by enhancing macrophage efferocytosis via MerTK-ERK-COX2 signaling
Source: Cell Death Discov. 2024 Sep 10;10:401. doi: 10.1038/s41420-024-02169-y (PMC11387478; doi:10.1038/s41420-024-02169-y)
Supplement: Supplementary file 1 — Supplementary figures and figure legends [file 41420_2024_2169_MOESM1_ESM.docx]

**Extracellular Vesicles Containing GAS6 Protect the Liver from Ischemia-Reperfusion Injury by Enhancing Macrophage Efferocytosis via MerTK-ERK-COX2 Signaling**

Miao Longyu^1*^, Yu Chaoqun^1*^, Guan Ge^2^, Luan Xiaoyu^1^, Jin Xiaoshuang^1^, Pan Meiqi^3^, Yang Yuzhen^3^, Yan Jiaoyang^4^, Chen Peng^1,5##^, Di Guohu^1,5,6#^

**Supplementary information**

**
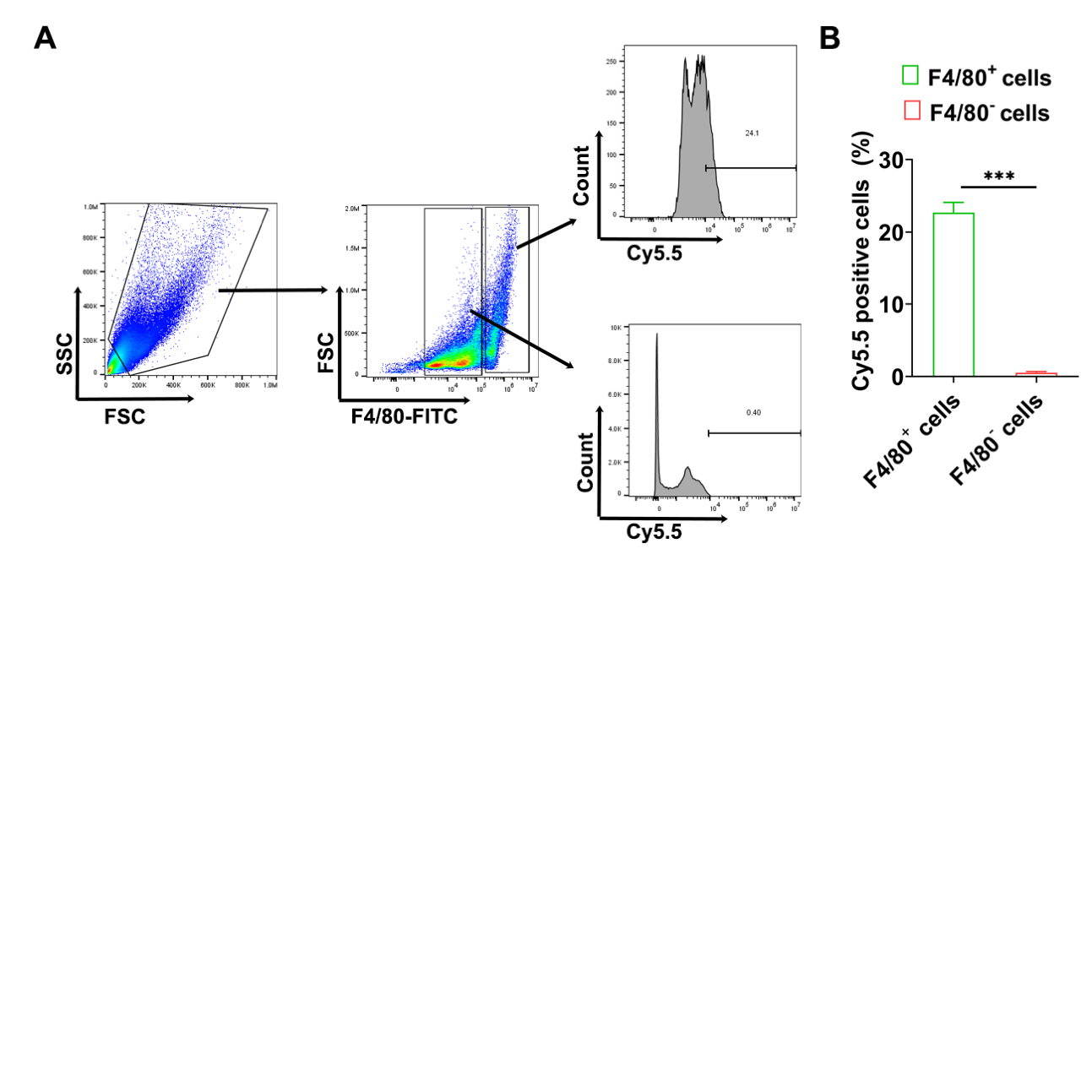
**

**Supplementary Figure 1.** Mice were injected with Cy5.5-stained MSC-EVs via tail vein, then liver tissue were collected, digested to single cells, and detected via flow cytometry at 6 hours post-surgery. (A) Gating strategy for single cells (irregular quadrilateral), macrophages (F4/80^+^, rectangle). (B) Quantification of Cy5.5 positive in different cell population.

**
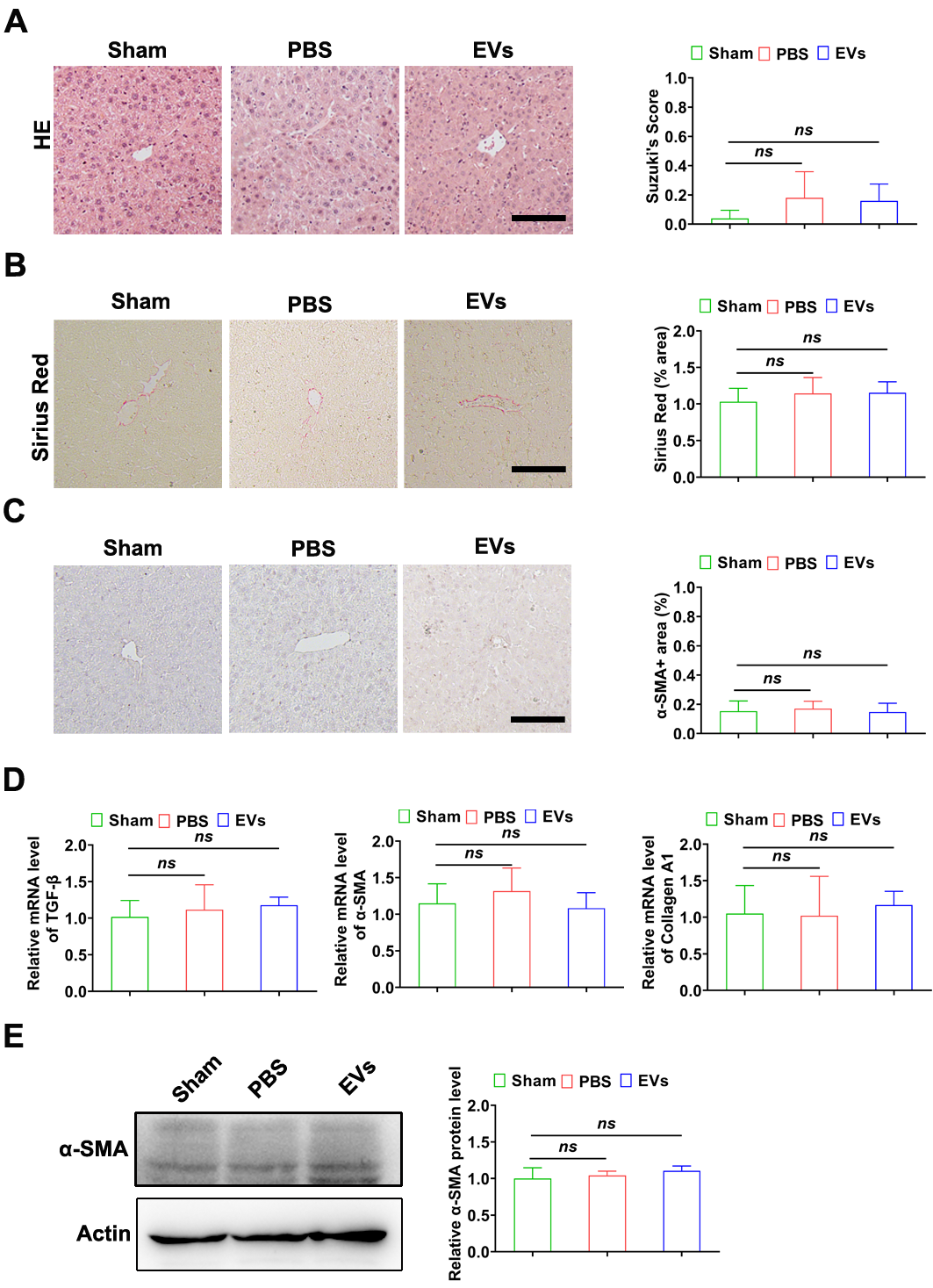
**

**Supplementary Figure 2.** MSC-EVs treatment have no long-term effects on liver fibrosis. (A-C) Representative images of liver sections stained with H&E, Sirius red, and α-SMA immunohistochemistry were obtained and quantified (n = 3 per group), Scale bar =100 µm. (D) The mRNA expression levels of TGF-β, COL1A1, and α-SMA in liver tissue were measured 7 days after the operation. (E) Western blot analysis was performed to determine the protein expression of α-SMA in liver tissue. Data were presented as the mean ± SD. ns: no significance.

**
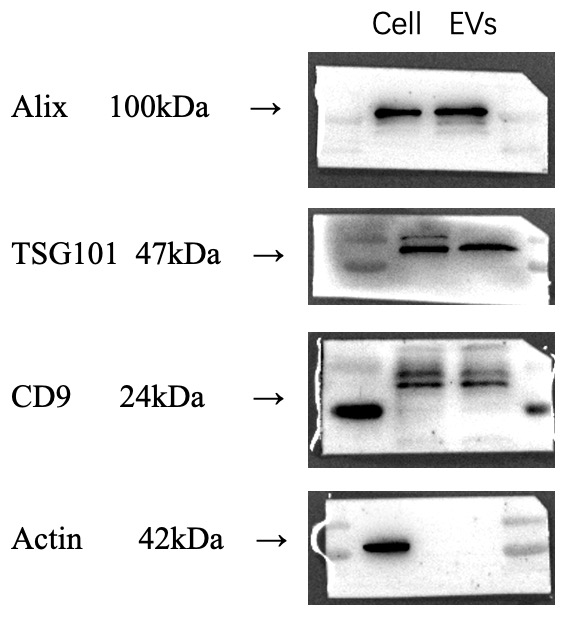
**

**Supplementary Figure 3.** **Full length gels of three independent western blots for Fig. 1C.** MSC-EVs were collected for western blot. Lane 1: MSCs; Lane 2:MSC-EVs;


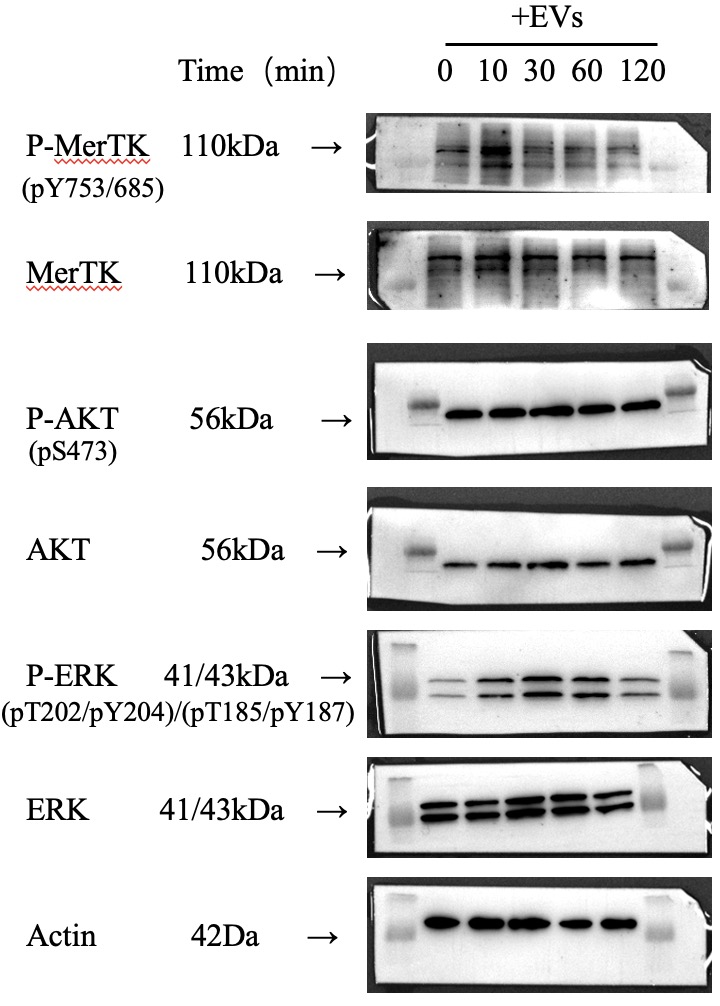


**Supplementary Figure 4. Full length gels of three independent western blots for Fig. 5B.** BMDMs pretreated with MSC-EVs for 0, 10, 30, 60, and 120 minutes. The cells were collected for western blot. Lane 1: 0 min; Lane 2: 10 min; Lane 3: 30 min; Lane 4: 60 min; Lane 5: 120 min.


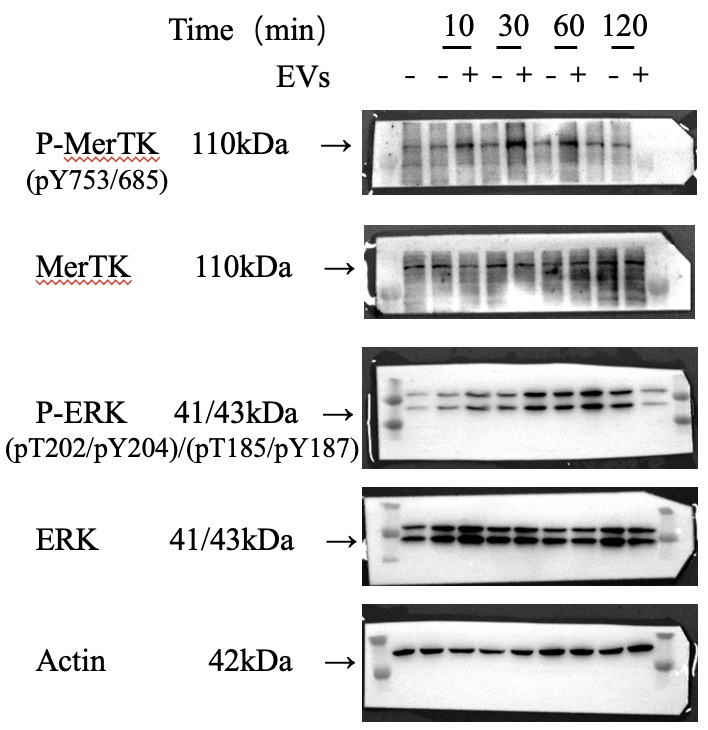


**Supplementary Figure 5. Full length gels of three independent western blots for Fig. 5E.** BMDMs pretreated or not with MSC-EVs for 120 minutes, then co-cultured with ACs for 0, 10, 30, 60, and 120 minutes and the cells were collected for western blot. Lanes as shown in the picture.


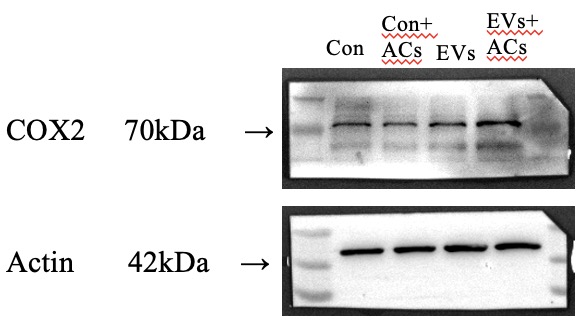


**Supplementary Figure 6. Full length gels of three independent western blots for Fig. 5F.** Lane 1: con; Lane 2: con+ ACs; Lane 3: EVs; Lane 4: EVs+ ACs.


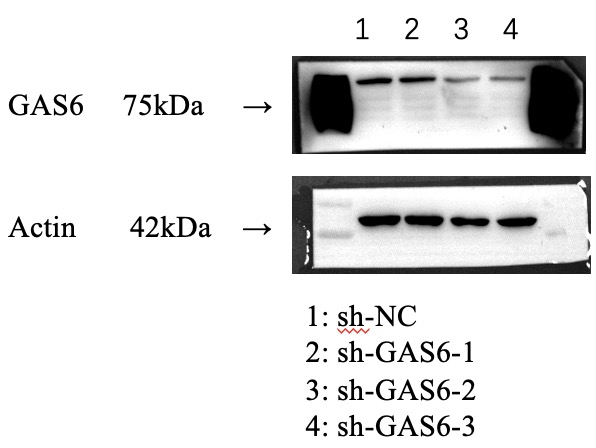


**Supplementary Figure 7. Full length gels of three independent western blots for Fig. 6A.** MSCs transfected by GAS6-specific lentivirus and the cells were collected for western blot. Lane 1: sh-NC group; Lane 2: sh-GAS6-1 group; Lane 3: sh-GAS6-2 group; Lane 4: sh-GAS6-3 group.


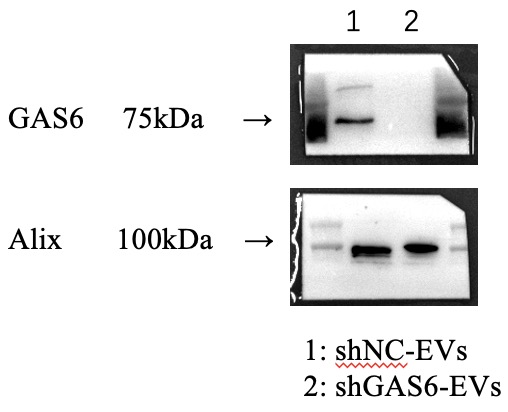


**Supplementary Figure 8. Full length gels of three independent western blots for Fig. 6B.** MSCs transfected by GAS6-specific lentivirus and the MSC-EVs were collected for western blot. Lane 1: shNC-EVs group; Lane 2: shGAS6-EVs group.


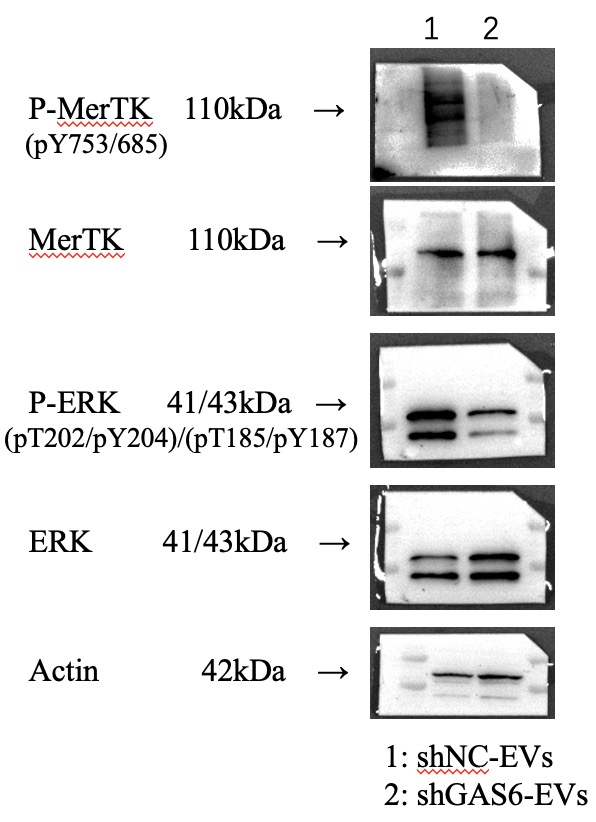


**Supplementary Figure 9. Full length gels of three independent western blots for Fig. 6D.** BMDMs pretreated with shNC-EVs or shGAS6-EVs for 120 minutes. The cells were collected for western blot. Lane 1: shNC-EVs; Lane 2: shGAS6-EVs.


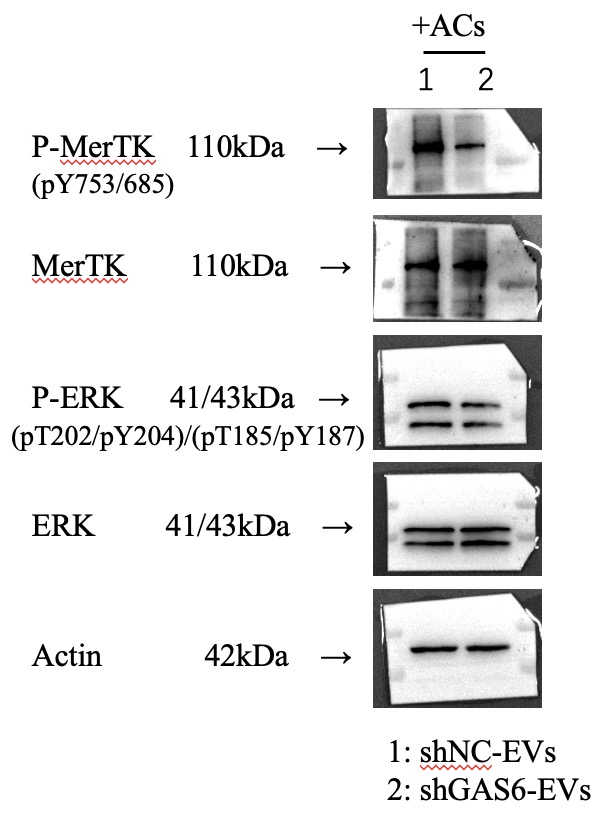


**Supplementary Figure 10. Full length gels of three independent western blots for Fig. 6E.** BMDMs pretreated with shNC-EVs or shGAS6-EVs, then co-cultured with ACs for 30 minutes and the cells were collected for western blot. Lanes as shown in the picture.


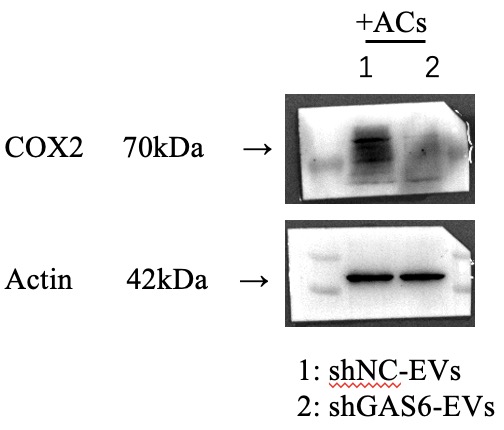


**Supplementary Figure 11. Full length gels of three independent western blots for Fig. 6F.** BMDMs pretreated with shNC-EVs or shGAS6-EVs, then co-cultured with ACs for 45 minutes, cells were collected 6 hours after removed the ACs.

Lane 1: shNC-EVs+ACs; Lane 2: shGAS6-EVs+ ACs.


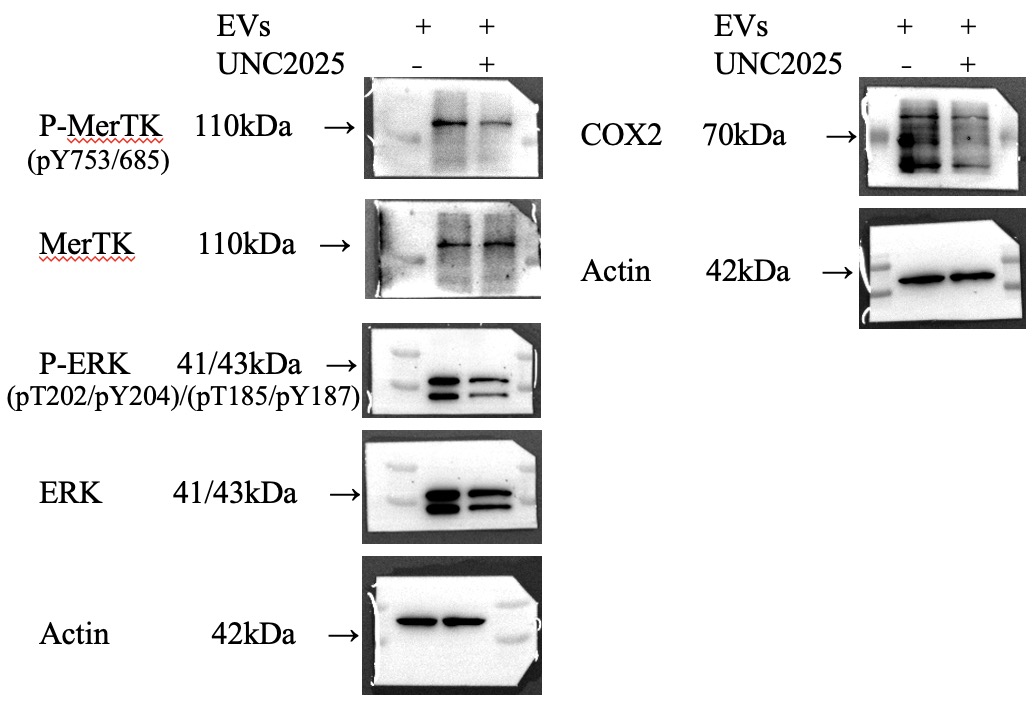


**Supplementary Figure 12. Full length gels of three independent western blots for Fig. 7B.** BMDMs pretreated with or without UNC2025 in the presence of MSC-EVs. Lanes as shown in the picture.


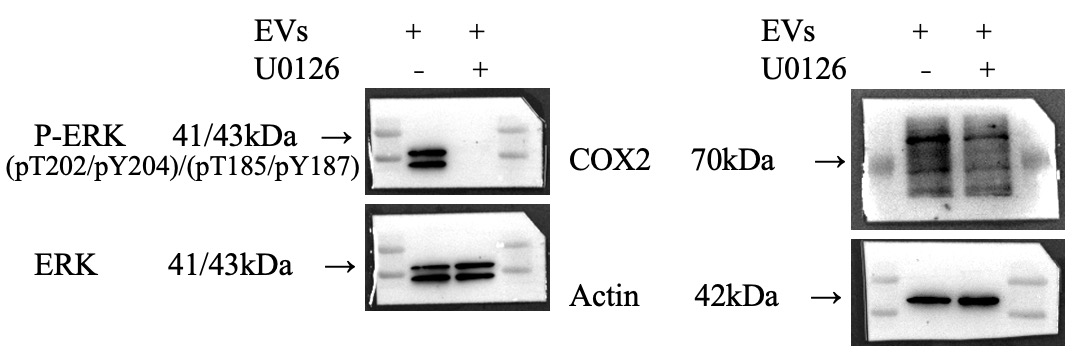


**Supplementary Figure 13. Full length gels of three independent western blots for Fig. 7E.** BMDMs treated with or without the ERK inhibitor U0126 in the presence of MSC-EVs. Lanes as shown in the picture.


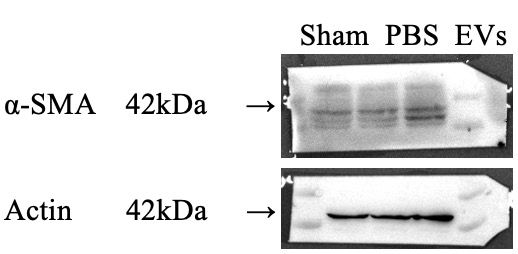


**Supplementary Figure 14. Full length gels of three independent western blots for Fig. 8E.** HIRI mice treated with PBS or MSC-EVs, and liver tissues were collected for western blot. Lane 1: sham group (a midline laparotomy incision without blocking blood); Lane 2: PBS group (PBS injected by tail vein); Lane 3: MSC-EVs group (MSC-EVs injected by tail vein).
